# Reversing EGFR Mediated Immunoescape by Targeted Monoclonal Antibody Therapy

**DOI:** 10.3389/fphar.2017.00332

**Published:** 2017-05-30

**Authors:** Fernando Concha-Benavente, Robert L. Ferris

**Affiliations:** ^1^Department of Otolaryngology, University of PittsburghPittsburgh, PA, United States; ^2^University of Pittsburgh Cancer InstitutePittsburgh, PA, United States

**Keywords:** EGFR, PD-L1, PD-1, HLA class I, Immunoescape, aerobic glycolysis, T cells

## Abstract

Uncontrolled growth is a signature of carcinogenesis, in part mediated by overexpression or overstimulation of growth factor receptors. The epidermal growth factor receptor (EGFR) mediates activation of multiple oncogenic signaling pathways and escape from recognition by the host immune system. We discuss how EGFR signaling downregulates tumor antigen presentation, upregulates suppressive checkpoint receptor ligand programmed death ligand (PD-L1), induces secretion of inhibitory molecules such as transforming growth factor beta (TGFβ) and reprograms the metabolic pathways in cancer cells to upregulate aerobic glycolysis and lactate secretion that ultimately lead to impaired cellular immunity mediated by natural killer (NK) cell and cytotoxic T lymphocytes (CTL). Ultimately, our understanding of EGFR-mediated escape mechanisms has led us to design EGFR-specific monoclonal antibody therapies that not only inhibit tumor cell metabolic changes and intrinsic oncogenic signaling but also activates immune cells that mediate tumor clearance. Importantly, targeted immunotherapy may also benefit from combination with antibodies that target other immunosuppressive pathways such PD-L1 or TGFβ and ultimately enhance clinical efficacy.

## Introduction

Cancer is characterized by uncontrolled cellular proliferation. The malignant oncogenic transformation of cells is caused by deleterious mutations of genes that control cell growth or activating mutations of those that favor cell division. However, unrestrained oncogenic proliferation is also caused by overexpression of molecules that are normally present in cells under homeostatic conditions, such as growth factor receptors. In this setting, one of the most studied oncogenic signaling pathways that has been characterized in many types of cancer is the ErbB/Her family of growth factor receptors that belong to the super family of receptor tyrosine kinases (RTK), given their capacity of phosphorylating tyrosine resides in their cytoplasmic tail and transduce extracellular signals through the activation of intracellular messengers (Linggi and Carpenter, 2006). The ErbB/Her family comprises four members: EGFR (ErbB1, HER1), ErbB2 (HER2), ErbB3 (HER3), and ErbB4 (HER4) (Linggi and Carpenter, 2006). The EGFR is considered a prototypical oncogenic growth factor receptor, since it activates multiple intracellular signaling transduction cascades including the mitogen activated protein kinase (MAPK), phosphatidylinositol-3 kinase (PI3K/AKT), Janus kinase/signal transduced, and activator of transcription (JAK/STAT) and protein kinase C (PKC) pathways (Marmor et al., 2004; Warren and Landgraf, 2006). Furthermore, several studies associate EGFR signaling with tumor progression of cancer, including breast, lung, head, and neck squamous cell carcinoma (HNSCC) and glioblastoma (Wada et al., 1990; Harari and Yarden, 2000; Blume-Jensen and Hunter, 2001). In addition to overexpression of wild type EGFR, some tumors also exhibit activating mutant forms such as glioblastoma, where a variant called EGFRvIII has been reported (Gan et al., 2013) or non-small cell lung cancer (NSCLC) where mutations in the gene encoding the EGFR kinase domain (T790M) have been associated with tumor resistance (Ohashi et al., 2013). Interestingly, other mutations such as the L858R mutation in exon 21 of the kinase domain of EGFR in NSCLC increased sensitivity to tyrosine kinase inhibition, however they did not improve clinical outcome (Peng et al., 2015).

The overactivation of EGFR downstream signaling pathways induces malignant transformation of tumor cells through increased cell proliferation and survival, resistance to growth inhibition or apoptosis and increased invasion and metastasis, capabilities that are a common denominator to the majority of tumors (Hanahan and Weinberg, 2000). Importantly, recent work has shown evidence for adding to this list of tumor transforming competences the downregulation of tumor cell immunogenicity which is key mediator for immune evasion. Indeed, the immune system plays a major role in tumor progression, immune mediated inflammation and the recruitment of immune infiltrating cells and their interaction with tumor cells and surrounding stromal cells forms an intricate cellular and molecular network called the tumor microenvironment (TME). Therefore, cancer progression not only depends on intrinsic growth factor signals that provide uncontrolled proliferation but also evasion of the host's antitumor immunity. In this context, the concept of cancer immunoediting originates, where tumor infiltrating immune cells specifically recognize highly immunogenic tumor cells at early stages of cancerous transformation and eliminate them, however a subset of these transformed cells survives elimination and enters the editing phase termed equilibrium. Subsequently, evolutionary pressure selects tumor cells that can progressively evade immune detection, leading to the escape and tumor growth (Schreiber et al., 2011). Recognizing that the immune system acknowledges the presence of cancer and sculpts its progress, underlines the importance of investigating and understanding the mechanisms by which these complex interactions occur and justifies the development of strategies to manipulate the host immune system in order to promote tumor control and elimination.

In this review we will discuss how tumor cell intrinsic oncogenic signals downstream the EGFR can lead to immunoescape by downregulating tumor cell immunogenicity such as diminishing HLA class I mediated antigen presentation or providing inhibitory signals, such as checkpoint inhibition mediated by programmed death ligand 1 (PD-L1), suppressive cytokines that induce an exhausted phenotype or modifying the extracellular milieu by upregulating concentrations of lactate. Additionally, we will also discuss how monoclonal antibody mediated EGFR inhibition can reverse immunoescape and induce activation of effector immune cell subsets such as CD8+ T cells and NK cells.

## EGFR mediated immunoescape, abnormal signals 1, 2, and 3

EGFR overexpression and overactivation of downstream pathways induces oncogenic transformation. In addition to its intrinsic oncogenic potential the EGFR also plays an important role in evasion of tumor immunosurveillance. Herein we present evidence to support the view that tumor immune evasion occurs by deregulating the three fundamental signals for an efficient immune activation: Signal 1, mediated by HLA class I dependent antigen presentation; signal 2, mediated by co-stimulatory receptor-ligand interaction and signal 3, mediated by secretion of immunostimulatory soluble cytokines.

### Aberrant signal 1

Proper antigen presentation is a major pre-requisite for appropriate T cell responses, especially because of the key role of this process in the generation of tumor antigen (TA)-specific adaptive immune responses (Meissner et al., 2005; Lopez-Albaitero et al., 2006). In the cancer setting, it has been recently reported that EGFR downregulates the expression of APM components and HLA class I via activation of protein phosphatase type 11 (PTNP11) best known as SHP2 which dephosphorylates signal transducer and activator of transcription 1 (STAT1). Less activated STAT1 translates into reduced expression of antigen presenting machinery (APM) and HLA class I dependent antigen presentation (Concha-Benavente et al., 2013; Leibowitz et al., 2013). Interestingly, inhibition or depletion of SHP2 in tumor cell lines or treatment with IFNγ induced upregulation of phosphorylated STAT1 (pSTAT1) and restored expression of HLA class I and APM components. Importantly, expansion of EGFR-specific CTL was noted upon an enhanced HLA class I restricted antigen presentation *in vitro* (Leibowitz et al., 2013). Likewise, SHP2-mediated pSTAT1 downregulation diminished the production of Th1 cytokines by tumor cells, since its inhibition induced increased secretion of interleukin-12 (IL-12) p35/p40 and IFNγ-dependent CXCR3 and CCR5 binding chemokines (Leibowitz et al., 2013). Furthermore, a second mechanism dependent on the MAPK pathway has been reported for downregulation of HLA class I and APM components downstream the EGFR, where activated SHP2 dephosphorylates GDP, inducing GTP-mediated RAS activation (Agazie and Hayman, 2003).

### Aberrant signal 2

An aberrant co-inhibitory signal 2 is represented by PD-L1/PD-1 pathway activation since recent studies have shown that this axis constitutes a major suppressive mechanism to evade immune activation and tumor clearance by downregulating T cell activation, proliferation, survival, cytotoxicity and cytokine release (Tseng et al., 2001; Dong et al., 2002). Moreover, inhibition of this pathway has proved to be clinically relevant since blocking antibodies against PD-1 or PD-L1 have demonstrated encouraging clinical activity in patients with metastatic melanoma, renal cell carcinoma (RCC), non-small cell lung cancer (NSCLC), and head and neck cancer (HNSCC), where PD-L1 tumor expression enriched for clinical responders (Brahmer et al., 2012; Topalian et al., 2012; Ferris et al., 2016). Importantly, a recent study demonstrated that PD-L1 expression in NSCLC cell lines is mediated by constitutively active mutant EGFR/KRAS-MAPK pathway (Akbay et al., 2013), whereas in the setting of HNSCC, overexpressed wild-type EGFR induced the expression of PD-L1 in a JAK2/STAT1 dependent manner. Curiously, although both cancers upregulate PD-L1 in an EGFR-dependent fashion, two different signaling pathways were involved JAK/STAT and MAPK pathways. This interesting finding could be explained by the unique biology of each cancer type, where mutant EGFR/KRAS would strongly activate MAPK pathway in NSLSC (Akbay et al., 2013). In contrast, HNSCC with a much lower EGFR/KRAS mutation burden (2%) (Stransky et al., 2011; McBride et al., 2014; Cancer Genome Atlas Netwrork, 2015) relies more on an overstimulated wild type EGFR/JAK2 pathway for oncogenic signaling (Concha-Benavente et al., 2016). Supporting these results is the recent finding by Zaretsky et al. where JAK2 inactivating mutations correlated with resistance to anti-PD-1 therapy in melanoma patients, in this setting we could speculate that tumor cells are sculpted by the immune system developing new strategies to evade anti-PD-L1/PD-1 pathway blocking therapy by downregulating JAK2-mediated expression of PD-L1 (Zaretsky et al., 2016). Interestingly, IFNγ, which restored of HLA class I expression and antigen presentation in tumor cells as discussed above is also a major inducer of PD-L1 expression as shown in multiple cancer types including HNSCC (Concha-Benavente et al., 2015), fibrosarcoma (Lee et al., 2005), glioblastoma (Han et al., 2009), and multiple myeloma cells (Liu et al., 2007). Notably, there appears to be a crosstalk between EGFR and IFNγ pathways regarding PD-L1 regulation at least in HNSCC, since EGFR blockade downregulated IFNγ-dependent PD-L1 expression (Concha-Benavente et al., 2016). Therefore, EGFR blockade may not only diminish the tumor cell intrinsic EGFR-induced PD-L1 upregulation but also the cell extrinsic IFNγ-mediated signals that have been associated with CD8+ T cell infiltration in the TME (Lyford-Pike et al., 2013; Topalian et al., 2015). Interestingly, EGFR signaling not only induces *de novo* expression of PD-L1 as shown in lung and head and neck cancer but also promotes stabilization of PD-L1 surface expression through glycosylation of its extracellular domain mediated by glycogen synthase kinase 3β (GSK3β) activation (Li et al., 2016). In this report authors show that EGFR may induce PD-L1 surface stabilization by inhibiting GSK3β in basal-like breast cancer cells. Moreover, gefitinib-mediated inhibition of EGFR destabilized PD-L1 surface expression in a GSK3β dependent fashion and enhanced tumor specific T cell immunity measured by CD8+ T cell IFNγ and granzyme B production. Importantly, in this setting EGFR inhibition increased efficacy of anti-PD-1 therapy in a syngeneic mouse model (Li et al., 2016).

### Aberrant signal 3

**Figure 1:**
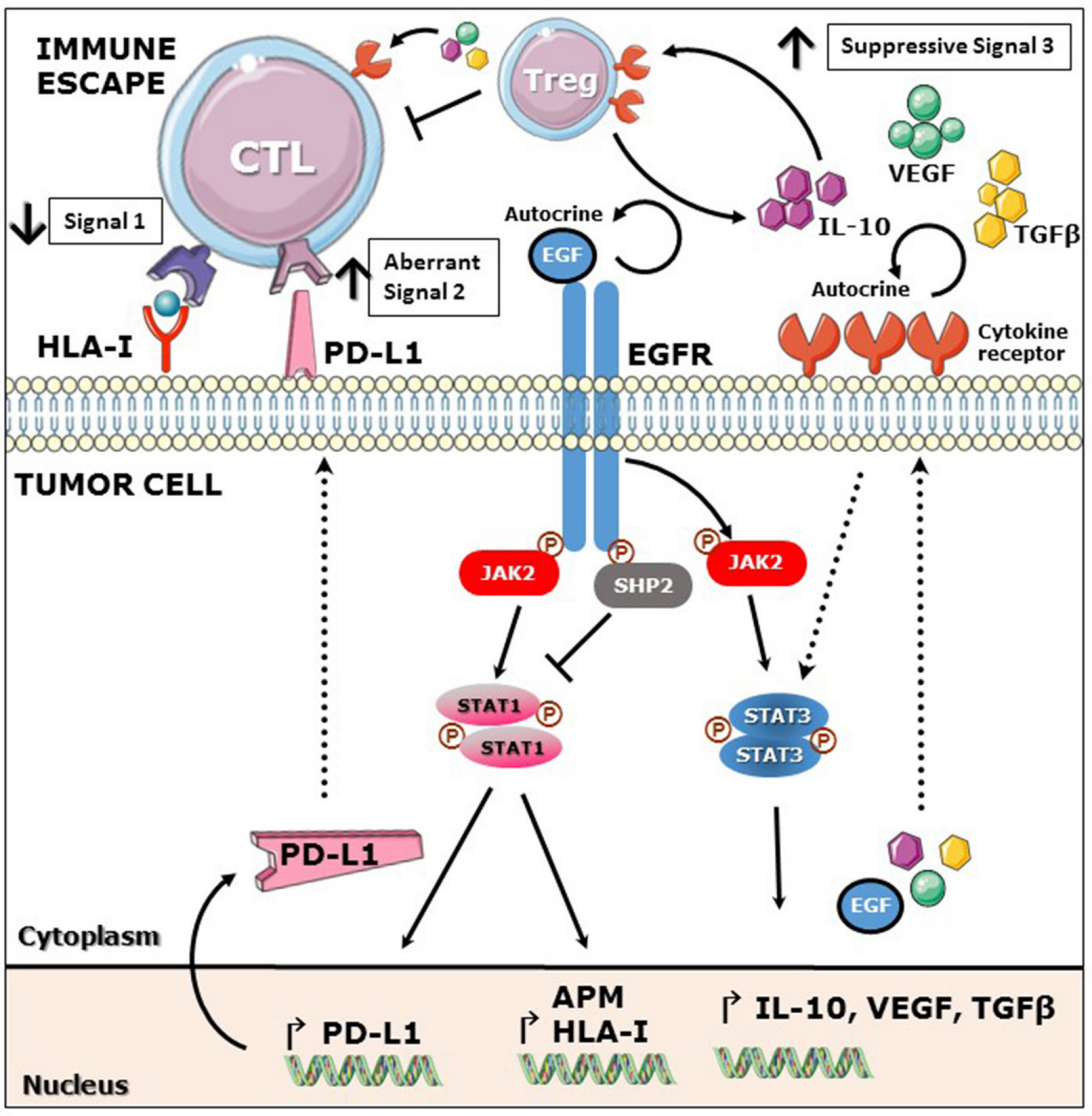
EGFR mediated immunoescape. EGFR stimulation induces activation of phosphatase SHP2 which decreases phosphorylation of STAT1 and subsequently expression of HLA class I and APM components. Additionally, EGFR mediated activation of JAK2-STAT1 induces expression of PD-L1. EGFR stimulation induces activation of STAT3 and production of immunosuppressive cytokines such as IL-10, VEGF and TGFβ which in turn induce Treg expansion and inhibition of CTL activation. Overall, EGFR signaling mediates downregulation of signal 1 and upregulation of suppressive signals 2 and 3, favoring escape from effector T cell recognition.

Signal 3 is required for an optimal effector T cell activation mediated by soluble cytokines and chemokines secreted in the milieu (Mescher et al., 2006). In the tumor microenvironment, the presence of suppressive cytokines promotes unresponsiveness of CD8+ T effector immune cell infiltrates and proliferation of suppressive cell subsets such as regulatory T cells (Treg), myeloid derived suppressive cells (MDSC) or tumor-associated macrophages (TAM) (Rabinovich et al., 2007; Burkholder et al., 2014). EGFR activation induces the constitutive activation of signal transducer and activator of transcription 3 (STAT3), a known oncogenic transcription factor (Grandis et al., 1998; Schrump and Nguyen, 2001; Kijima et al., 2002). STAT3 plays a major role in promoting tumor immune evasion since it not only has opposite effects to immunostimulatory STAT1 but also induces the secretion of immunosuppressive soluble cytokines that induce a tolerant TME (Wang et al., 2004; Kortylewski et al., 2005). Previous studies showed that STAT3 mediates the expression of vascular endothelial growth factor (VEGF), IL-6, and IL-10 in many cancer types, which induce T cell tolerance through inhibition of DC differentiation and maturation (Gabrilovich et al., 1996; Yang and Lattime, 2003). In addition, IL-6, IL-10, and VEGF activate STAT3 in tumor infiltrating immune cells, providing a feed forward mechanism for STAT3 activation in the tumor microenvironment. An important immunosuppressive cytokine, transforming growth factor beta (TGFβ), is secreted by many cancers including melanoma, breast and colon cancer and is known to prevent cytotoxic T cell (CTL) expansion and activation (Mooradian et al., 1990), TGFβ and IL-10 are involved in the generation of regulatory T cells (Treg) which in turn inhibit CD8^+^ T cell activation and IFNγ secretion (Nishikawa et al., 2005). Additionally, Tregs are an important source of TGFβ and IL-10, which once secreted to the TME further propagate the immunosuppressive signals (Figure 1; Zorn et al., 2006; Larmonier et al., 2007).

## EGFR-mediated regulation of tumor cell metabolism and immunoescape

Tumor cells in contrast to most normal cells require large amounts of energy to support an increased rate of uncontrolled division. In this setting, one distinctive characteristic of cancer cells is to accelerate glucose degradation a metabolic process called glycolysis. Unlike normal tissues where glycolysis is limited by oxygen levels, tumor cells can reprogram their metabolic machinery to increase glycolysis and generate large amounts of lactate as a byproduct, a process that has been termed “aerobic glycolysis” and recognized as one of the new hallmarks of cancer (Hanahan and Weinberg, 2011; Ward and Thompson, 2012). Importantly, the lactate rich immunosuppressive TME impairs cytolytic functions of CD8+ T cells *in vitro* as well as their proliferation and cytokine production (Fischer et al., 2007). Interestingly, previous reports from more than a decade ago linked EGFR stimulation with enhanced aerobic glycolysis and lactate production in cancer cells including breast cancer (Kaplan et al., 1990; Baulida et al., 1992), however the mechanism by which the EGFR enhances glycolysis and lactate secretion was just recently reported. Lim et al. showed that EGFR stimulation in triple negative breast cancer (TNBC) cells induces activation of hexokinase 2 (HK2) and pyruvate kinase M2 (PKM2) an enzymatic isoform that is only expressed in embryonic cells and cancer cells. These two enzymes regulate the first and last steps of glycolysis respectively, and promote the accumulation of glycolytic intermediaries that ultimately enhance tumor cell proliferation. Interestingly, accumulation of the glycolytic intermediary fructose 1, 6 bisphosphate (F16BP) in TNBC cells via the activation of HK2 and PKM2 was found to directly engage the EGFR and enhance its phosphorylation and activation, thereby providing a positive feedback loop which ultimately increased lactate production and inhibition of CTL activity. Importantly, dual EGFR and glycolysis inhibition effectively suppressed TNBC cell proliferation and tumor growth (Lim et al., 2016). Similarly, work done by Makinoshima et al. showed that EGFR signaling maintained aerobic glycolysis in lung adenocarcinoma (LAD) cells and enhanced the extracellular acidification rate (ECAR) via lactate secretion. Moreover, specific EGFR inhibition with small molecule inhibitors gefitinib and erlotinib downregulated glucose transporter 3 (GLUT3) expression and that of genes involved in the pentose phosphate pathway (PPP) and pyrimidine synthesis. Overall, this study suggests that EGFR signaling regulates global metabolic pathways in EGFR-mutated LAD cells and may be an important target to reverse tumor cell–derived metabolic immunosuppression (Makinoshima et al., 2014).

Finally, EGFR activation has also been shown to upregulate hypoxia inducible factor 1 alpha (HIF-1α), a crucial molecule that reprograms cellular metabolism from oxidative phosphorylation to aerobic glycolysis. Interestingly, EGFR inhibition with monoclonal antibody cetuximab downregulated expression of HIF-1α and lactate dehydrogenase A (LDH-A), a crucial enzyme regulating the conversion of pyruvate to lactate. Interestingly, this inhibition only occurred in cetuximab-sensitive but not in cetuximab-resistant HNSCC cell lines, data that argues in favor of a metabolic immunoescape mechanism to EGFR inhibition of HNSCC cells mediated by lactate acidification of the tumor milieu (Lu et al., 2013). Overall these findings support the view that EGFR contributes to immune escape not only by downregulating tumor cell immunogenicity (signal 1) or providing aberrant signals 2 and 3 but also inducing an acidic, lactate rich milieu that impairs an effective adaptive antitumor immunity.

## Activation of anti-tumor immunity by anti-EGFR targeted therapy

EGFR activation occurs upon ligand binding, therefore disruption of such interaction by targeted receptor-blocking specific agents is a logical strategy to shutdown not only its oncogenic metabolic shift and proliferative signaling but also the pathways involved in providing aberrant signals 1, 2, and 3. In this setting, small molecule inhibitors have been developed for inhibiting EGFR-induced phosphorylation of tyrosine residues of its cytoplasmic tail, two of such tyrosine kinase inhibitors (TKI) have been approved for clinical use, gefitinib and erlotinib (Stamos et al., 2002; Yun et al., 2007). In addition to small molecule inhibitors, two monoclonal antibodies (mAbs) targeting the EGFR have been approved for clinical use, cetuximab, an IgG1 chimeric mAb and panitumumab, a humanized IgG2 mAb, both inhibiting EGFR signaling to the same extent (Trivedi et al., 2016). Importantly, one added benefit of using EGFR blocking antibodies is that their interaction with EGFR extracellular domain may also induce endocytosis and degradation of the receptor (Wieduwilt and Moasser, 2008).

However, arguably the most important benefit of EGFR blocking mAbs is the activation of immune system effector cells via interaction of the antibody's Fc portion with the Fcγ receptors (FcγR) expressed on the surface of effector cells. In the case of cetuximab, this is supported by the observations that even though it interfered with growth signals, it did not induce cell death, probably because of activation of alternative survival pathways in cancer cells independent of the EGFR. Interestingly, cetuximab induced tumor cell death only when NK cells were added to *in vitro* co-cultures (Lopez-Albaitero and Ferris, 2007; Lopez-Albaitero et al., 2009; Taylor et al., 2009), therefore the major cetuximab antitumor effect seems to be immune mediated. Studies later revealed that cetuximab IgG1 framework allows its interaction with FcγRIIIA (CD16) on NK cells. Binding of cetuximab to CD16 on NK cells triggers a lytic process called antibody-dependent cellular cytotoxicity (ADCC) and IFNγ secretion. Moreover, NK cell-derived IFNγ mediates cross talk with DCs inducing their maturation and HLA class I antigen presentation which subsequently induce clonal expansion of EGFR-specific CD8^+^ T cells (Rafiq et al., 2002; Harbers et al., 2007; Banerjee et al., 2008; Marechal et al., 2010; Lee et al., 2011). Interestingly, the clinical activity of cetuximab is effective by increasing patient survival either as monotherapy or when added to radiation or platinum-based chemotherapy (Bonner et al., 2006; Vermorken et al., 2007, 2008). In addition to enhancing NK mediated cytotoxicity which is likely its most conspicuous effect, it has been recently shown that it could also activate neutrophils and mediate ADCC against EGFR expressing tumor cells via interaction with FcγRIIa, interestingly, this cytotoxic effect was FcγRIIIa genotype-dependent (Trivedi et al., 2016). On the other hand, panitumumab has shown less clinical efficacy than cetuximab (Mesia et al., 2015), this result may be explained by a less potent NK cell activation induced by panitumumab given its IgG2 framework, furthermore, its monocyte activation capability may be innocuous to EGFR expressing tumor targets given that monocytes are not capable of mediating ADCC. These preclinical findings are further supported by clinical data where patients treated with cetuximab had higher EGFR-specific cytotoxic CD8+ T cells when compared with those treated with panitumumab (Trivedi et al., 2016). Interestingly, it has also been reported that cetuximab-mediated EGFR blockade induced Treg expansion in head and neck cancer patients which correlated with resistance to cetuximab therapy (Jie et al., 2015), such Treg expansion was induced partially by DC maturation and T cell receptor stimulation in the presence of TGFβ. Interestingly, these *in vitro* expanded Tregs suppressed NK cell cytotoxicity against tumor cells providing evidence for Treg mediated immunosuppression in the tumor microenvironment.

## Conclusions

Several studies support the view that tumors evolve intrinsic mechanisms to evade immune recognition. In this review we presented evidence for the ErbB/Her receptor family member the EGFR as an important driver of immunoescape by downregulating crucial immune activating signals 1, 2, and 3 and by inducing a metabolic shift of tumor cells to aerobic glycolysis and lactate secretion into the tumor microenvironment. Increasing our understanding of the mechanisms that tumor cells use to escape immunosurveillance will allow strategies to reverse EGFR mediated immune escape. Importantly, targeted mAb immunotherapy has the advantage of not only suppressing tumor intrinsic suppressive signals but also activating tumor infiltrating immune system cells which has shown clinical efficacy in many types of cancer. However, the majority of EGFR-overexpressing tumors have a complex genetic background with a significant level of compensatory oncogenic pathways regulating cell metabolism, proliferation, trafficking, and survival. Therefore, combination therapy not only targeting the EGFR but also other important molecules that regulate cellular immune responses in the TME such as the PD-L1/PD-1 axis or TGFβ and other metabolic immunosuppressive byproducts such as lactate should enhance immune responses and improve their clinical efficacy.

## Author contributions

FC wrote the manuscript and draw the figure, RF reviewed the manuscript and approved the final version.

### Conflict of interest statement

The authors declare that the research was conducted in the absence of any commercial or financial relationships that could be construed as a potential conflict of interest. The reviewer ZA and handling Editor declared their shared affiliation, and the handling Editor states that the process nevertheless met the standards of a fair and objective review.
